# Targeted exome sequencing reveals novel *USH2A* mutations in Chinese patients with simplex Usher syndrome

**DOI:** 10.1186/s12881-015-0223-9

**Published:** 2015-09-16

**Authors:** Hai-Rong Shu, Huai Bi, Yang-Chun Pan, Hang-Yu Xu, Jian-Xin Song, Jie Hu

**Affiliations:** Department of Otolaryngology, Taizhou Central Hospital, Taizhou University School of Medicine, No 999, Donghai Rd., Taizhou, Zhejiang 318000 China; Department of Otolaryngology, Taihe People’s Hospital, No 158, Shengli Rd., Taihe, Jiangxi 343700 China; Department of Otolaryngology, the Forth Affiliated Hospital of Shihezi University School of Medicine, Beier Rd., Shihezi, Xinjiang 843000 China; Department of Ophthalmology, Taizhou Central Hospital, Taizhou University School of Medicine, No 999, Donghai Rd., Taizhou, Zhejiang 318000 China

## Abstract

**Background:**

Usher syndrome (USH) is an autosomal recessive disorder characterized by hearing impairment and vision dysfunction due to retinitis pigmentosa. Phenotypic and genetic heterogeneities of this disease make it impractical to obtain a genetic diagnosis by conventional Sanger sequencing.

**Methods:**

In this study, we applied a next-generation sequencing approach to detect genetic abnormalities in patients with USH. Two unrelated Chinese families were recruited, consisting of two USH afflicted patients and four unaffected relatives. We selected 199 genes related to inherited retinal diseases as targets for deep exome sequencing. Through systematic data analysis using an established bioinformatics pipeline, all variants that passed filter criteria were validated by Sanger sequencing and co-segregation analysis.

**Results:**

A homozygous frameshift mutation (c.4382delA, p.T1462Lfs*2) was revealed in exon20 of gene *USH2A* in the F1 family. Two compound heterozygous mutations, IVS47 + 1G > A and c.13156A > T (p.I4386F), located in intron 48 and exon 63 respectively, of *USH2A,* were identified as causative mutations for the F2 family. Of note, the missense mutation c.13156A > T has not been reported so far.

**Conclusion:**

In conclusion, targeted exome sequencing precisely and rapidly identified the genetic defects in two Chinese USH families and this technique can be applied as a routine examination for these disorders with significant clinical and genetic heterogeneity.

## Background

Usher syndrome (USH) is the most prevalent cause of hereditary deafness-blindness in humans [1]. It occurs in approximately 1 of 25,000 persons worldwide. USH is divided into clinical subtypes I, II, or III, based on severity and progression of vision and hearing loss [2]. Patients with Usher I have congenital deafness and begin to lose their vision early in life. They also exhibit difficulty balancing due to vestibular problems. Patients with Usher II exhibit congenital hearing loss that is mild-moderate in low tones and moderate to severe at higher frequencies. They generally have a normal vestibular system. Patients with Usher III are not born deaf, but experience a gradual loss of both their hearing and vision. Usher II is the most common form of USH, and may account for more than half of all patients with USH [3]. Such complex clinical heterogeneity often precludes an accurate clinical diagnosis. Hearing loss in USH is usually caused by impairment of inner ear structures such as hair cells, which transmit sound and motion signals to the brain. Vision loss results from the degeneration of photoreceptors in the retina [4].

Due to the genetic heterogeneity of USH, molecular diagnosis using traditional Sanger sequencing is quite laborious and expensive [5, 6]. To date, 12 genes (*CDH23, CEP250, CIB2, CLRN1, DFNB31, GPR98, HARS, MYO7A, PCDH15, USH1C, USH1G* and *USH2A*) have been reported to be responsible for Usher syndrome and three additional loci have been mapped. *USH2A*, the major pathogenic gene for Usher II, has 72 exons, the entire region of which is scattered with implicated mutations. Next-generation sequencing (NGS) technology has recently been demonstrated to be superior than Sanger for screening of hundreds of target genes at a given time [7]. In this study, we adopted an NGS-based capture technique to reveal genetic defects in two USH families and aimed to evaluate this method as a routine diagnostic tool for USH patients.

## Methods

### Patient recruitment

This study was performed in accordance with the tenets of the Declaration of Helsinki and the ethics committee of Taizhou Central Hospital, and written informedconsent was obtained from all study subjects. Two unrelated Chinese families, including two affected and four unaffected individuals, were enrolled. Clinical diagnosis of Usher syndrome was based on examination of audiologic function, visual acuity, fundus photography and optical coherence tomography (OCT). Typical initial symptoms of USH include hearing loss, vision acuity impairment, night blindness, and bone spicule-like pigmentation and attenuation of retinal vessels in fundus.

### Sample preparation

Peripheral blood was collected from all subjects and used for DNA extraction using a kit (TIANGEN, Beijing) according to the manufacturer’s instructions. DNA yield was quantified with Nanodrop 2000 (Thermal Fisher Scientific, DE).

### Targeted gene capture and sequencing

A solution-based sequence capture custom enrichment kit was used (GenCap, MyGenostics, Beijing), including 199 disease genes [8]. A minimum of 3 ug DNA was extracted per proband, and fragmented into 200–300 bp DNA libraries according to the manufacturer’s protocol. After end-repair and adapter ligation, target libraries were hybridized with the panel and enriched using the Illumina Exome Enrichment protocol. Purified and enriched libraries were then sequenced using an Illumina HiSeq 2000 Sequencer.

### Bioinformatics analysis

After HiSeq 2000 re-sequencing, raw sequencing reads were first filtered by the Solexa QA package [9] and then by the cutadapt program (https://pypi.python.org/pypi/cutadapt). High quality reads were aligned to the reference genome sequence (hg19) by using Burrows-Wheeler Aligner and duplications were removed by using Sequence Alignment/Map tools [10]. Only unique aligned reads were kept for further analysis. Single-nucleotide variants (SNVs) and insertions or deletions (InDels) were identified by GATK software [11], and filtered to eliminate low quality SNVs or those with strand bias or proximity to InDels. Remaining variants were functionally annotated across the genome by ANNOVAR. The pathogenicity of nonsynonymous variants was then evaluated by four algorithms, PolyPhen2, SIFT, PANTHER and Pmut, as described previously [12].

### Expanded validation

DNA samples of the parents of two probands were used to validate all likely pathogenic variants by using conventional Sanger sequencing, and to perform familial segregation analyses whenever possible. Purified PCR products were cycle-sequenced on an ABI 3500 Genetic Analyzer (Applied Biosystems, CA). The results of Sanger sequencing were analyzed by Mutation Surveyor (Softgenetics, PA).

## Results

### Clinical findings

**Fig. 1:**
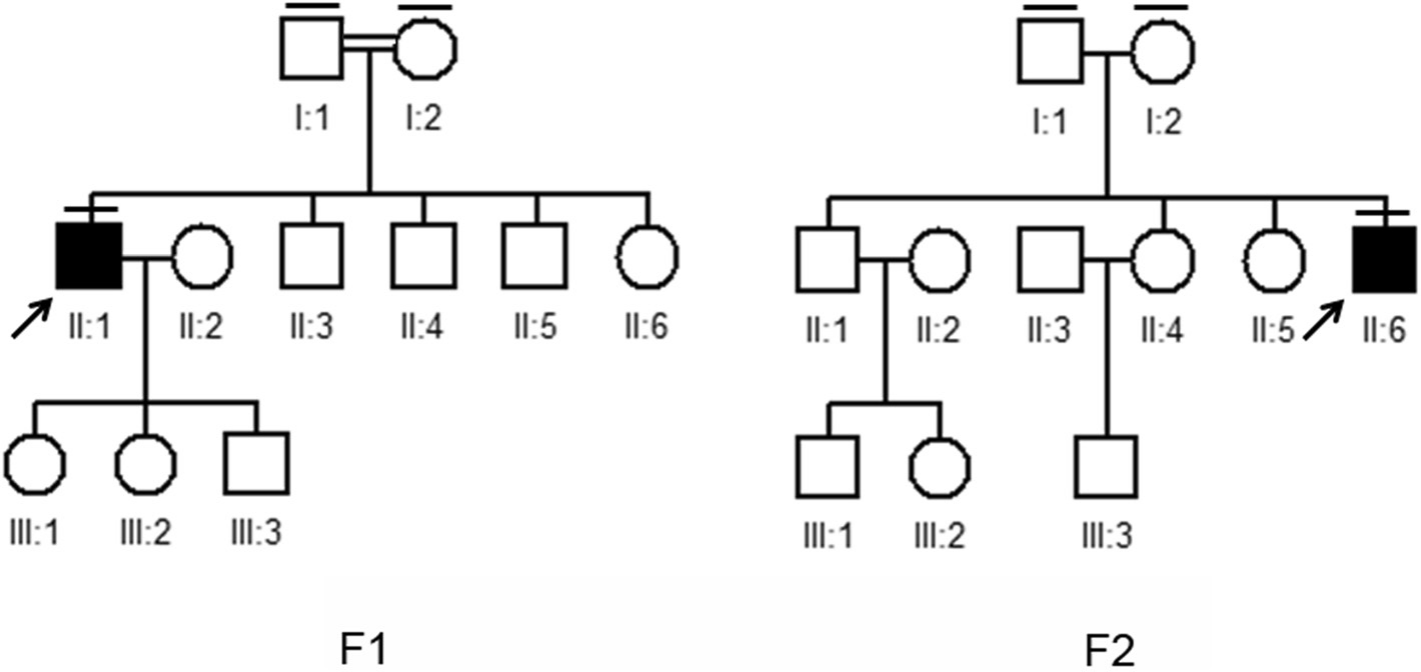
Pedigrees of Usher syndrome in this study. Closed symbols represent affected patients and open symbols indicate unaffected subjects. The bar over the symbol indicates subjects examined in this study

**Fig. 2:**
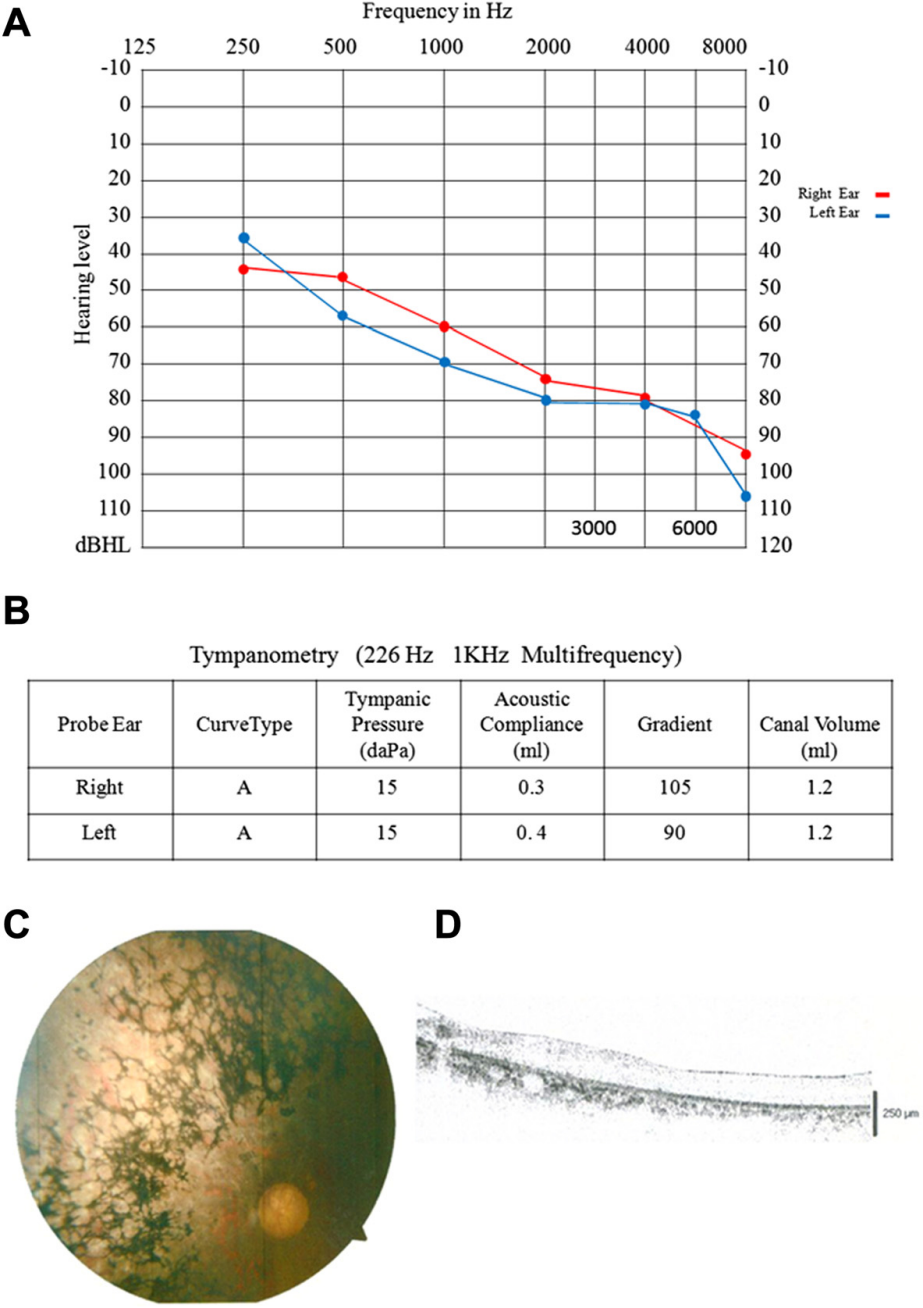
Clinical examination. **a** Pure tone audiogram showed bilateral downward-sloping moderate to severe hearing loss in one patient with Usher syndrome (F1-II-1); **b** Tympanometry examination demonstrated the normal functions of middle ear and eardrum in the F1-II-1 proband; **c** Fundoscopy of the F1-II-1 patient revealed symptoms typical of retinitis pigmentosa. **d** OCT of the F1-II-1 patient showed that retinal thickness was diminished

The two families enrolled in this study had USH sporadic cases, and two individuals (F1-I-1, F1-I-2) in family F1 were consanguineous (Fig. 1). The on-set age of both probands are about 15 years old. In family F1, the proband (F1-II-1) was 50 years old and suffered night blindness since adolescence. His visual deterioration had been progressive and at the time of the study he was nearly completely blind. He had no vestibular system problems. Analysis of pure tone audiogram testing showed bilateral moderate to severe hearing loss, moderate at low sound frequencies sloping to severe at higher tested frequencies. Tympanometry examination demonstrated the normal functions of middle ear and eardrum. Upon ophthalmologic examination, fundus photography showed significant bone-spicule pigmentation in the retinal periphery, attenuated retinal arteries and waxy pallor of the optic nerve, all typical signs of retinitis pigmentosa. Results of the OCT test clearly displayed extremely thin and disorganized inner and outer photoreceptor segments (Fig. 2). Collectively, this clinical evidence suggested a diagnosis of Usher II for this proband. The patient, F2-II-6 from family F2 was only 20 years old and exhibited symptoms similar to that of proband F1-II-1 (data not shown). However, the severity of his symptoms was relatively mild, likely due to his younger age.

### Candidate mutations identified by targeted exome sequencing

We performed targeted exome sequencing of two proband samples from the USH families. The average sequencing depths and coverage of the target regions were 300 reads and 98.0 %, respectively. The coverage of targeted exons for >10 reads was 90.7 % and >20 reads was 81.6 %. After processing of raw data through the BWA program, variants were called and annotated using GATK. We identified numerous non-synonymous variants by the GATK program. Any variants that were reported in the HapMap 28 and the SNP release of the 1000 Genome Project with a MAF > 0.05 were removed. To evaluate the pathogenicity of the variants, we used bioinformatics tools to analyze potential impacts on the function or structure of the encoded proteins. By employing this stepwise analyses approach, we obtained a list of candidate mutations for the two probands. Finally, there were only one homozygous and two heterozygous variants in *USH2A* in these two pedigrees, respectively.

### Expanded familial validation and Sanger sequencing confirmation

**Fig. 3:**
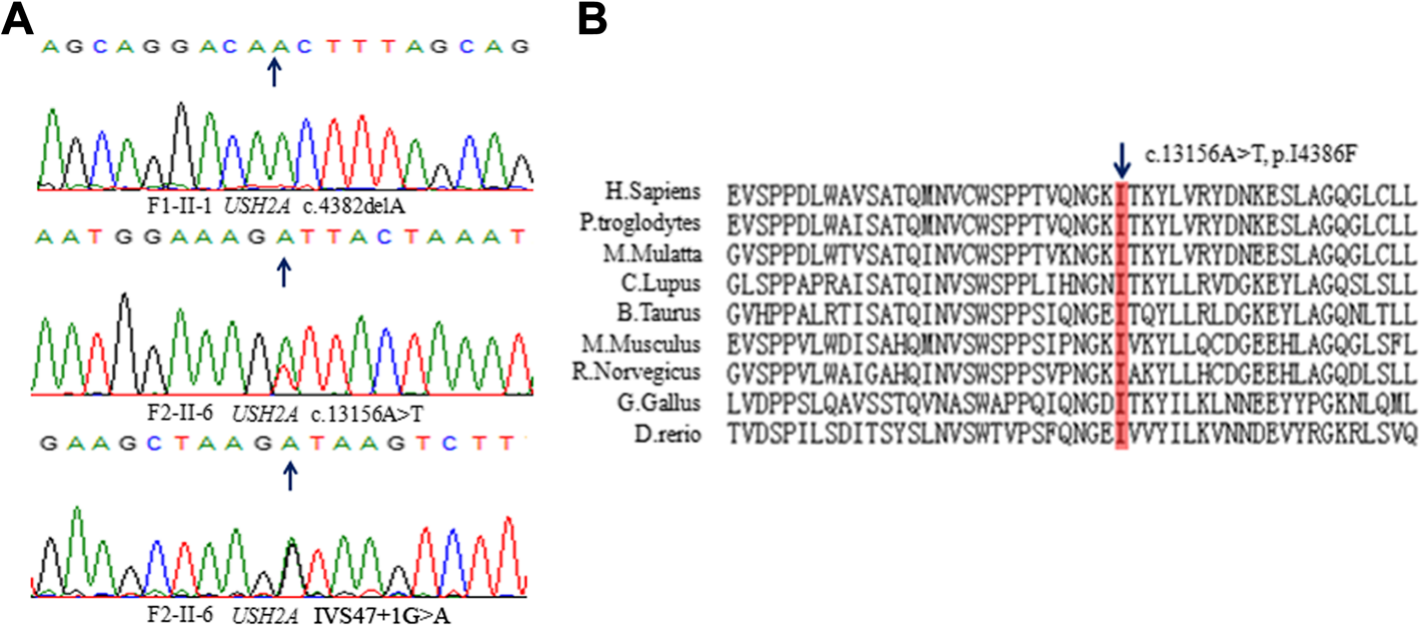
Chromatography and conservation analyses of the mutations. **a** Sanger sequencing validated mutations harbored by the two probands. **b** Amino acid sequence alignments of the areas of novel mutation, in various species

Results from targeted exome sequencing of samples from the two probands indicated that they probably had defects in the *USH2A* gene. To validate the sequencing results, we used Sanger sequencing to screen the same mutation sites in their parents’ DNA. In family F1, we confirmed a heterozygous frameshift mutation (c.4382delA, p.T1462Lfs*2) in exon 20 of *USH2A*. In family F2, the unaffected father was found to have a heterozygous splice site mutation (IVS47 + 1G > A) in intron 48 of *USH2A* and the unaffected mother had a novel heterozygous mutation (c.13156A > T) in exon 63 of *USH2A*. The affected proband, F2-II-6, harbored both compound heterozygous *USH2A* mutations (IVS47 + 1G > A, c.13156A > T) from his parents (Fig. 3).

### Analysis of *USH2A* mutations

The frameshift mutation (c.4382delA, p.T1462Lfs*2) and the splicing site mutation (IVS47 + 1G > A) found here has been previously reported in a previous study [8]. The missense variant (c.13156A > T) has not been reported in the 1000 Genomes database or in any other single nucleotide polymorphism database. This mutation caused substitution of phenylalanine for isoleucine at amino acid position 4386 and was speculated by prediction software to effect protein functions. In this study, the pathogenicity of missense was then evaluated by four algorithms, PolyPhen2, SIFT, PANTHER and Pmut. All these four algorithms showed the p.I4386F was damaging. It is located in a highly conserved domain of *USH2A*, when compared to other species (Fig. 3). In summary, we successfully identified causative mutations in two USH families, by combining Sanger sequencing and co-segregation analysis.

## Discussion

Usher syndrome (USH) is the most common cause of hereditary deafness–blindness of humans. The typical symptoms are hearing loss and visual dysfunction, with differing severities in each subtype [13, 14]. Clinical and genetic heterogeneity of USH makes it difficult to accurately diagnose this disease at a molecular level by conventional direct sequencing. Targeted exome sequencing is an efficient and low-cost technique for sequencing hundreds of potential target genes at one time. In this study, we employed a comprehensive strategy to screen USH patients for mutation in large group of target genes responsible for inherited retinal degeneration. We performed target exome sequencing on two probands and successfully identified causative mutations by combining familiar validation and co-segregation analyses.

In these two Chinese USH families, mutations in the *USH2A* gene were identified to be possibly disease-causing. The missense heterozygous SNV (c.13156A > T) was a novel mutation located in a highly conserved region in *USH2A*. The protein encoded by the *USH2A* gene, usherin, is critical for proper development of the inner ear and retina, which helps explain the incidence of disease-associated hearing and vision loss. Our findings expanded the spectrum of known *USH2A* mutations and reflected the significant implication of *USH2A* in Usher syndrome (Table 1).

**Table 1:** Identified mutations in *USH2A* gene

Family	Proband	Mutation	Type	Amino Acid	Reference
F1	II-1	c.4384delA	Homo	p.T1462Lfs*2	Reported
F2	II-3	IVS47 + 1G > A	Hetero	Splice site	Reported
F2	II-3	c.13156A > T	Hetero	p.I4386F	Novel

## Conclusions

The technology we employed in this study will increase the accuracy of efficiency by which patients with retinal dystrophy such as USH, and retinitis pigmentosa receive diagnosis and prognosis. For those patients and their family members who are at high risk of disease, an accurate and timely clinical diagnosis can help them obtain personalized therapy and professional advice on concerns relating to marriage and procreation.

## Abbreviations

USH: Usher syndrome
SNVs: Single-nucleotide variants
Indels: Insertions or deletions
OCT: Optical coherence tomography
PCR: Polymerase chain reaction
NGS: Next-generation sequencing
